# Erfahrungen mit Gefährdungssituationen in Psychiatrie und Psychotherapie bei Patienten mit extremistischer Einstellung

**DOI:** 10.1007/s00115-023-01469-5

**Published:** 2023-03-22

**Authors:** Thea Rau, Sophia Mayer, Anna Heimgartner, Marc Allroggen

**Affiliations:** Klinik für Kinder- und Jugendpsychiatrie und Psychotherapie, Universitätsklinikum Ulm, Steinhövelstraße 5, 89075 Ulm, Deutschland

**Keywords:** Extremismus, Krankenbehandlung, Sicherheit, Vernetzung, Ärzt:innen, Extremism, Health care, Safety, Networking, Doctors

## Abstract

**Hintergrund und Fragestellung:**

Gefährdungssituationen im Zusammenhang mit der Krankenbehandlung von Personen mit vermuteter extremistischer Einstellung spielen insbesondere in den letzten Jahren vermehrt eine Rolle. Eine Befragung von Ärzt:innen und Psychotherapeut:innen soll Aufschluss über konkrete Gefährdungslagen bei diesen Patient:innen geben.

**Material und Methoden:**

Mittels einer anonymen Online-Befragung, welche 16 Haupt- und bis zu 95 weitere Fragen umfasste, wurden insgesamt 364 Angehörige von Heilberufen zur allgemeinen Situation und zu Patient:innen sowie Angehörigen mit vermuteter extremistischer Einstellung befragt.

**Ergebnisse:**

17,5 % der Teilnehmenden sind Ärzt:innen, 72,1 % nichtärztliche Psychotherapeut:innen bzw. in Ausbildung (47,7 % arbeiten ausschließlich in einer Klinik, 34,2 % in einer niedergelassenen Praxis). Insgesamt haben 57,7 % der Teilnehmenden schon einmal Patient:innen mit vermuteter extremistischer Einstellung behandelt (46,7 % behandelten Angehörige). 27,6 % wurden im Rahmen der Krankenbehandlung mit Selbstgefährdungssituationen konfrontiert (30,1 % bei Angehörigen), 49,5 % mit Situationen der Fremdgefährdung (18,3 % bei Angehörigen), bei denen sie sich häufig im Umgang damit nicht sicher gefühlt haben. 20,3 % der Fachkräfte informierten die Sicherheitsbehörden, nicht ganz die Hälfte empfand diesen Kontakt als eher nicht/gar nicht hilfreich (45,5 % bei Angehörigen). Kontakt zu anderen Stellen, zum Beispiel auch zu spezialisierten Fachberatungsstellen für Deradikalisierung, hatte die Mehrheit nicht. Ärzt:innen erlebten Gefährdungssituationen häufiger als nichtärztliche Psychotherapeut:innen. Ein Vergleich zwischen Fachkräften aus der Klinik und niedergelassenen Praxis zeigt keine bedeutsamen Unterschiede.

**Diskussion:**

Die Studie konnte zeigen, dass Extremismus und damit einhergehend Gefährdungslagen ein wichtiges Thema in der Krankenbehandlung sind und Ärzt:innen und Psychotherapeut:innen darauf gut vorbereitet sein sollten. Für die Zukunft wäre eine Vernetzung mit Stellen der Extremismusprävention wichtig und eine gute Kooperation mit den Sicherheitsbehörden wünschenswert.

## Abstract (en)

**Background and objectives:**

Dangerous situations in connection with the treatment of persons with suspected extremist attitudes have become increasingly relevant. A survey of physicians and psychotherapists is intended to provide information about concrete dangerous situations among these patients.

**Materials and methods:**

By means of an anonymous online survey, which comprised 16 main questions and up to 95 additional questions, a total of 364 health professionals were asked about the general situation and about patients, as well as relatives with suspected extremist attitudes.

**Results:**

In all, 17.5% of the participants were physicians, 72.1% psychotherapists. 47.7% work exclusively in a hospital, 34.2% in a private practice. A total of 57.7% of the participants have already treated patients with suspected extremist attitudes (46.7% treated relatives); 27.6% were confronted with situations of self-endangerment (30.1% in the case of relatives), 49.5% with situations of danger to others (18.3% in the case of relatives), in which they often did not feel safe. Altogether, 20.3% of the professionals informed security authorities, and not quite half found this contact to be comparatively unhelpful/not at all helpful (45.5% among relatives). The majority of the participants had no contact to other agencies, such as specialized counselling centres for deradicalization. Physicians experienced the situations of endangerment more often than non-medical psychotherapists. A comparison between professionals from hospitals and private practices shows no significant differences.

**Discussion:**

The study was able to show that extremism and the associated dangerous situations are an important topic in the treatment of patients and that physicians and non-medical psychotherapists should be well prepared. Networking with extremism prevention agencies and good cooperation with security authorities would be important and desirable for the future.

## Hintergrund und Fragestellung

In den letzten Jahren wurde vermehrt die Rolle von Psychotherapeut:innen und Ärzt:innen in der Extremismusprävention diskutiert, vor allem auch in Bezug auf die öffentliche Sicherheit [1, 4, 30]. Anlass waren unter anderem terroristische Anschläge und Gewaltdelikte mit extremistischem Hintergrund von Einzeltätern, bei denen es vor der Tat Kontakte mit psychiatrischen Einrichtungen gab [17, 21]. Für eine bessere Gefahrenabwehr und Früherkennung von Gefährdungslagen wurde in diesem Zusammenhang gefordert, dass Ärztinnen und Ärzte bei der Behandlung solcher Patient:innen frühzeitig die Sicherheitsbehörden kontaktieren sollten [18]. Dabei ist jedoch unklar, wie häufig Fachkräfte aus Heilberufen überhaupt mit dem Thema Extremismus im Rahmen der Krankenbehandlung konfrontiert werden und wie sie dabei mit möglichen Gefährdungslagen umgehen.

Die vorliegenden Befunde zum Zusammenhang von psychischen Erkrankungen und extremistischen Einstellungen und Gewalttaten müssen differenziert betrachtet werden. Mehreren Studien zufolge zeigen terroristische Einzeltäter (sogenannte „lone actors“) über die klassischen Phänomenbereiche hinweg (v. a. Rechtsextremismus, Islamismus) eine erhöhte Prävalenz für psychische Erkrankungen (u. a. aus dem schizophrenen Formenkreis, wahnhafte Störungen, Autismusspektrumstörungen, Suchterkrankungen), sowohl im Vergleich zur Allgemeinbevölkerung [10] als auch im Vergleich zu Mitgliedern von extremistischen Gruppen [2, 6, 7, 11, 14, 25]. Bei Untersuchungen von Personen mit extremistischer Einstellung oder Personen, die in extremistischen Gruppen agieren, lässt sich jedoch grundsätzlich keine überdurchschnittlich hohe Prävalenz für psychische Störungen wie affektive Störungen, Suchterkrankungen, Persönlichkeitsstörungen oder Erkrankungen aus dem schizophrenen Formenkreis im Vergleich zur Allgemeinbevölkerung feststellen, jedoch allgemein Fälle von psychischen Erkrankungen dieser Art, depressive Störungen und auch Suizidalität [31]. Daneben zeigen sich in vielen Untersuchungen überproportional häufig Auffälligkeiten im psychosozialen Bereich (z. B. Substanzmittelgebrauch, Probleme bei der Lebensführung im Alltag), die zur Entwicklung von Radikalisierungsprozessen beigetragen haben können [3, 7–9, 22, 26]. Über alle Studien hinweg, die Kindheitserlebnisse mituntersuchten, lassen sich zudem hohe Raten von belastenden Kindheitserfahrungen wie Vernachlässigung oder Missbrauchserfahrungen finden („adverse childhood experiences“ [ACE]; [5, 13, 28]).

Betrachtet man die Zusammenhänge zwischen psychischen Erkrankungen und dem Ausüben von Gewaltstraftaten, ist zunächst festzuhalten, dass psychisch erkrankte Personen nicht generell häufiger Gewaltstraftaten ausüben als gesunde Menschen, insbesondere wenn sie eine entsprechende Behandlung erhalten [16]. Allerdings gibt es psychische Erkrankungen, die mit einer erhöhten Gewaltbereitschaft assoziiert sind, wie beispielweise schwere wahnhafte Störungen, Schizophrenien oder Suchterkrankungen [23, 33]. Untersuchungen zeigen dabei, dass in Fällen von psychischen Erkrankungen das Risiko für selbst- und fremdgefährdendes Verhalten bei einem großen Teil der Betroffenen deutlich gesenkt werden kann, wenn diese behandelt werden, vor allem bei Erkrankungen aus dem schizophrenen Formenkreis [27].

Fasst man die Studienlage also zusammen, kann nur bei sogenannten „lone actors“ von einer erhöhten Prävalenz von bestimmten psychischen Störungen ausgegangen werden, nicht jedoch bei Personen mit extremistischer Einstellung und bei Personen, die in extremistischen Gruppen eingebunden sind. Ob aber psychische Störungen Radikalisierungsprozesse überhaupt begründen können, ist eher zweifelhaft, vielmehr scheinen gemeinsame Risikofaktoren für psychische Erkrankung und Radikalisierungsprozesse eine Rolle zu spielen.

Wissenschaftliche Untersuchungen, die auch die Perspektive von Ärzt:innen und psychologischen Psychotherapeut:innen zu deren Erfahrungen mit dem Thema im Rahmen der Behandlung berücksichtigen, fehlen bislang, ebenso, ob sie dabei mit Selbst- und Fremdgefährdungssituationen von Patientinnen und Patienten mit extremistischer Einstellung konfrontiert werden und ob es dabei zu einem Kontakt mit den Sicherheitsbehörden kommt.

Ziel der vorliegenden deutschlandweiten Online-Befragung war es daher, Angehörige von Heilberufen in Kliniken und niedergelassenen Praxen zu ihren Erfahrungen mit Gefährdungsaspekten von Patientinnen und Patienten mit extremistischer Einstellung in Psychiatrie und Psychotherapie und im Umgang mit Gefährdungssituationen zu befragen.

## Studiendesign und Untersuchungsmethode

Von März 2022 bis Juli 2022 wurde eine anonyme Online-Befragung mittels der Umfragesoftware „unipark“ unter (approbierten und in Ausbildung befindlichen) nichtärztlichen (Kinder- und Jugendlichen‑)Psychotherapeut:innen sowie Fachärzt:innen und Weiterbildungsassistent:innen in den Fachgebieten Psychiatrie und Psychotherapie, psychosomatische Medizin und Psychotherapie und Kinder- und Jugendpsychiatrie und Psychotherapie sowie sonstigen Angehörigen von Heilberufen durchgeführt. Zur Rekrutierung wurden Landespsychotherapeutenkammern und -ärztekammern, Fachgesellschaften, Berufsverbände, forensische Kliniken und Ausbildungsinstitute um eine Verbreitung des Befragungslinks unter den eigenen Mitgliedern gebeten. Für die Studie liegen ein Datenschutzkonzept nach der DSGVO und dem Landesdatenschutz Baden-Württemberg sowie eine Proband:inneninformation zur ausführlichen Aufklärung und Information der Teilnehmenden vor. Die Studie fällt nicht unter § 15 der Berufsordnung für Ärzt:innen in Baden-Württemberg. Ein Votum der Ethikkommission war daher nicht erforderlich.

Die Online-Befragung umfasste insgesamt 16 Hauptfragen und bis zu 95 weitere Fragen, die lediglich dann präsentiert wurden, wenn eine der Hauptfragen mit „ja“ beantwortet wurde. In der vorliegenden Arbeit wird über die beiden Hauptfragen zur Häufigkeit der Behandlung von Patient:innen mit extremistischer Einstellung oder ihren Kontaktpersonen, z. B. von Angehörigen, berichtet. Bei den beiden Hauptfragen konnte die Auswahloption „gar nicht“ (Filter), „einmal“, „mehr als einmal“ ausgewählt werden. Bei Angaben zu mehreren Personen konnte die Anzahl der Patient:innen per Freitextangabe eingegeben werden. Weitere Unterfragen wurden zum Zeitpunkt des ersten entsprechenden behandelten Patienten (in Jahren) gestellt: ob es zu Situationen der Selbst- und/oder Fremdgefährdung (z. B. Suizidgedanken, Äußerungen von Tatplänen, Gewaltanwendung) im Rahmen der Krankenbehandlung kam (Antwortoption von 1 = „gar nicht“, 2 = „einmal“, 3 = „mehr als einmal“, 4 = „regelmäßig“), wie sicher sich die-/derjenige im Umgang mit dieser/diesen Behandlungssituation(en) gefühlt hat (Antwortoption von 1 = „gar nicht“, 2 = „eher nicht“, 3 = „eher“, 4 = „sehr“), ob es erforderlich war, die Polizei oder andere Vertreter:innen der Sicherheitsbehörden bei einer Selbst- und/oder Fremdgefährdung hinzuzuziehen (1 = „gar nicht“, 2 = „einmal“, 3 = „mehr als einmal“, 4 = „regelmäßig“) und wenn ja, ob dieser Kontakt als hilfreich wahrgenommen wurde (1 = „gar nicht“, 2 = „eher nicht“, 3 = „eher“, 4 = „sehr“). Zusätzlich wurde abgefragt, mit welchen Stellen während der Behandlung Kontakt aufgenommen wurde. Dabei wurde eine Auswahl von vier Möglichkeiten vorgegeben (Fachberatungsstelle für Deradikalisierung, Hotline/Beratungsstelle „Radikalisierung“ des Bundesamts für Migration und Flüchtlinge [BAMF]), Präventionsprojekt bzw. -angebot, Sicherheitsbehörde (z. B. Polizei, Landeskriminalamt). Weitere Stellen konnten per Freitextangaben unter der zusätzlichen Auswahlkategorie für sonstige Stellen ergänzt werden, ebenfalls konnte die Auswahl „mit keiner“ (Stelle) getroffen werden. Zusätzlich wurden demografische Daten abgefragt, beispielweise die Berufsbezeichnung, der aktuelle Tätigkeitsort, Tätigkeitsjahre im Beruf (i. S. v. Berufserfahrung) und das Geschlecht der Befragten. Den Fragen ging eine Definition voraus, nach der unter einer extremistischen Einstellung ideologische, politische oder religiös motivierte Überzeugungen verstanden werden, die sich außerhalb bzw. am Rande einer freiheitlich-demokratischen Grundordnung bewegen und auch in Verbindung mit illegalen oder gewalttätigen Handlungen stehen können, z. B. Gewaltanwendungen auf einer Demonstration [4]. Beispielhaft genannt wurden linksextremistische Strömungen, der Rechtsextremismus, neu formierte Bewegungen wie z. B. die „Querdenken“-Bewegung oder der islamistische Extremismus. Die Befragten sollten im Rahmen der Beschreibung von Patient:innen, bei denen sie eine extremistische Einstellung vermuteten, auch einschätzen, welchem Phänomenbereich sie die Einstellung dieser Patient:innen zuordnen würden. Dazu wurden sechs Antwortmöglichkeiten vorgegeben mit Mehrfachauswahlmöglichkeiten (Islamismus, andere religiös begründete Ideologie, Rechtsextremismus, Linksextremismus, „Querdenken“-Bewegung, Sonstiges) und es bestand die Möglichkeit, explizit anzugeben, wenn keine Zuordnung gemacht werden konnte.

## Statistische Analysen

Für die Datenanalyse wurden die Befragungsdaten in die Auswertungssoftware IBM SPSS Statistics 27 importiert. Die einzelnen Antworten zu Gefährdungssituationen und Kontaktsituationen (bspw. mit Behörden) wurden für die Befragung zu Patient:innen mit vermuteter extremistischer Einstellung sowie für Angehörige zu einer Situation zusammengefasst und dichotomisiert zu „es kam jemals zu einer Selbstgefährdungssituation“ (1) bzw. „es kam jemals zu einer Fremdgefährdungssituation“ (1) und „es kam nie dazu“ (2). Darüber hinaus wurden zur Bewertung von Gruppenunterschieden zwischen Angaben von nichtärztlichen Psychotherapeut:innen und Ärzt:innen sowie zwischen in einer niedergelassenen Praxis und klinisch tätigen Teilnehmenden (Zusammenfassung der Auswahloptionen: Klinik/Abteilung für Psychiatrie und Psychotherapie, für Kinder- und Jugendpsychiatrie/Psychotherapie, für forensische Psychiatrie und Psychotherapie, für forensische Kinder- und Jugendpsychiatrie und Psychotherapie, für psychosomatische Medizin und Psychotherapie sowie Konsiliar- und/oder Liaisondienst, Rehaklinik, psychiatrische/psychosomatische Institutsambulanz) jeweils Chi-Quadrat-Tests durchgeführt. Bei den Vergleichen zwischen Ärzt:innen und nichtärztlichen Psychotherapeut:innen wurden 38 Teilnehmende ausgeschlossen, welche sonstige Berufsbezeichnungen angaben oder innerhalb der Auswahloptionen sowohl Ärzt:innen auswählten als auch angaben, nichtärztliche Psychotherapeut:innen zu sein (*n* = 2), da bei diesen Personen keine eindeutige Zuordnung zu einer der beiden Vergleichsgruppen möglich war. Ebenfalls wurden 66 Teilnehmende bei den Vergleichen ausgeschlossen, die sonstige Tätigkeitsbereiche angaben oder gleichzeitig in einer Praxis und einer Klinik arbeiten (*n* = 12), da auch bei diesen Personen keine eindeutige Zuordnung für einen Vergleich möglich war. Das Signifikanzniveau wurde auf 0,05 festgelegt.

## Ergebnisse

### Stichprobenbeschreibung

Von 364 Teilnehmenden (75,6 % weiblich, 24,1 % männlich, 0,3 % divers) liegen verwertbare Fragebögen aus der Online-Befragung vor. Die Berufserfahrungen der Teilnehmenden liegen bei durchschnittlich 13,28 Jahren (SD ± 12,11 Jahre). 17,5 % (*n* = 64) gaben an, Ärztin/Arzt zu sein, davon befinden sich 28,1 % (*n* = 18) in Weiterbildung und 72,1 % (*n* = 263) gaben an, nichtärztliche Psychotherapeut:innen im Kinder- oder Erwachsenenbereich zu sein bzw. den Beruf anzustreben. 10,4 % (*n* = 38) gaben sonstige Berufsbezeichnungen an oder arbeiteten als ärztliche Psychotherapeut:innen. 47,7 % (*n* = 174) arbeiteten ausschließlich in einer Klinik und 34,2 % (*n* = 125) waren ausschließlich in einer Praxis tätig, bei 18,1 % (*n* = 66) wurden sonstige Tätigkeitsbereiche oder sowohl Tätigkeiten in der Klinik als auch in einer Praxis angegeben (siehe Tab. 1).

**Table 1:** 

		Anzahl (*n*)	%
*Tätigkeit/Ausbildung* Mehrfachnennungen möglich	Psychologische:r Psychotherapeut:in	118	32,2
	Psychologische:r Psychotherapeut:in in Ausbildung	97	26,6
	Kinder- und Jugendlichenpsychotherapeut:in	26	7,1
	Kinder- und Jugendlichenpsychotherapeut:in in Ausbildung	36	9,9
	Fachärzt:in für Psychiatrie und Psychotherapie	31	8,5
	Ärzt:in in Weiterbildung zur/zum Fachärzt:in für Psychiatrie und Psychotherapie	10	2,7
	Fachärzt:in für psychosomatische Medizin und Psychotherapie	5	1,4
	Ärzt:in in Weiterbildung zur/zum Fachärzt:in für psychosomatische Medizin und Psychotherapie	2	0,5
	Fachärzt:in für Kinder- und Jugendpsychiatrie und Psychotherapie	14	3,8
	Ärzt:in in Weiterbildung zur/zum Fachärzt:in für Kinder- und Jugendpsychiatrie und Psychotherapie	6	1,6
	Sonstiges, z. B. Fachärzt:in für Allgemeinmedizin, Fachpsycholog:in für Rechtspsychologie, Sozialarbeiter:in etc.	44	12,1
*Aktueller Tätigkeitsort* Mehrfachnennungen möglich	Klinik/Abteilung für Psychiatrie und Psychotherapie	59	16,2
	Klinik/Abteilung für Kinder- und Jugendpsychiatrie/Psychotherapie	29	7,9
	Klinik/Abteilung für forensische Psychiatrie und Psychotherapie	27	7,4
	Klinik/Abteilung für forensische Kinder- und Jugendpsychiatrie und Psychotherapie	1	0,3
	Klinik/Abteilung für psychosomatische Medizin und Psychotherapie	23	6,3
	Konsiliar- und/oder Liaisondienst	7	1,9
	Rehabilitationsklinik	24	6,6
	Psychiatrische/psychosomatische Institutsambulanz (einschließlich Ausbildungsambulanzen)	52	14,2
	Inhaber:in/Angestellte:r in Praxis	137	37,5
	Sonstiges, z. B. Bewährungs- und Gerichtshilfe, JVA, Maßregelvollzug, Gutachten, sozialpsychiatrische Dienste etc.	69	18,9

### Patient:innen mit extremistischer Einstellung

Von den 364 Teilnehmenden an der Studie gaben 210 (57,7 %) an, Patient:innen mit extremistischer Einstellung behandelt zu haben, davon gaben 73 (34,8 %) an, einmal einen Fall behandelt zu haben, 137 (65,2 %) berichteten, mehr als einmal Patient:innen behandelt zu haben. Im Mittel wurden 5,29 (SD = 7,76) behandelte Patient:innen angegeben (Range 1–50). 170 (46,7 %) Teilnehmende gaben an, Angehörige von Personen mit extremistischer Einstellung behandelt zu haben, davon einmal 56 (32,9 %) und mehr als einmal 114 (67,1 %). Insgesamt hatten 247 Teilnehmende (67,9 %) Erfahrungen mit Patient:innen mit extremistischer Einstellung und/oder Angehörigen.

Die Teilnehmenden beschrieben bis zu fünf Patient:innen näher. Von diesen insgesamt 242 beschriebenen Patient:innen wurden 98 (40,5 %) dem Bereich Rechtsextremismus, 80 (33,1 %) der „Querdenken“-Bewegung, 29 (12 %) dem Islamismus, 15 (6,2 %) dem Linksextremismus und 13 (5,4 %) einer anderen religiös begründeten Ideologie zugeordnet. Es konnten Mehrfachangaben gemacht werden. Bei 29 Patient:innen (12 %) wurde keine der Optionen gewählt. Bei 7 (0,4 %) wurde explizit die Option „Ich kann es nicht zuordnen“ gewählt.

Im Mittel wurden 6,19 (SD = 9,21) Angehörige von extremistischen Personen insgesamt behandelt (Range 1–70). Durchschnittlich gaben die Befragten 6,91 (SD = 7,28) Jahre Erfahrung in der Behandlung von Patient:innen mit extremistischer Einstellung an und 7,10 (SD = 7,92) Jahre in der Behandlung von Angehörigen von extremistischen Personen.

### Situationen der Selbst- und Fremdgefährdung

Von den Befragten, die bereits Patient:innen mit extremistischer Einstellung behandelten, gaben 58 (27,6 %) an, mit Selbstgefährdungssituationen (z. B. Suizidgedanken) in Verbindung mit deren Behandlung konfrontiert gewesen zu sein. Von denen, die Angehörige behandelten, berichteten 51 (30,1 %) von solchen Situationen. 104 (49,5 %) Teilnehmende gaben an, mit Situationen der Fremdgefährdung bei der Behandlung von Patient:innen konfrontiert gewesen zu sein, z. B. durch Äußerungen von Tatplänen oder durch direkte Gewaltanwendungen, 31 (18,3 %) berichteten solche Situationen im Zusammenhang mit der Behandlung von Angehörigen (siehe Tab. 2).

**Table 2:** 

**Frage**	**Gar nicht** **1**	**Einmal** **2**	**>** **Einmal** **3**	**Regelmäßig** **4**	**k.** **A.**
*Selbst*gefährdungssituation bei *Patient:innen* mit extremistischer Einstellung (*n* = 210)	150 (71,4 %)	22 (10,5 %)	33 (15,7 %)	3 (1,4 %)	2 (1,0 %)
*Selbst*gefährdungssituation bei *Angehörigen* (*n* = 170)	116 (68,2 %)	21 (12,4 %)	28 (16,5 %)	2 (1,2 %)	3 (1,8 %)
*Fremd*gefährdungssituation bei *Patient:innen* mit extremistischer Einstellung (*n* = 210)	105 (50,0 %)	41 (19,5 %)	53 (25,2 %)	10 (4,8 %)	1 (0,5 %)
*Fremd*gefährdungssituation bei *Angehörigen* (*n* = 170)	136 (80,0 %)	12 (7,1 %)	17 (10,0 %)	2 (1,2 %)	3 (1,8 %)
**Frage**	**Gar nicht** **1**	**Eher nicht** **2**	**Eher** **3**	**Sehr** **4**	**k.** **A.**
Sicherheit im Umgang mit *Selbst*gefährdung bei *Patient:innen* mit extremistischer Einstellung (*n* = 60)	0 (0,0 %)	13 (21,7 %)	37 (61,7 %)	10 (16,7 %)	0 (0,0 %)
Sicherheit im Umgang mit *Selbst*gefährdung bei *Angehörigen* (*n* = 54)	2 (3,7 %)	11 (20,4 %)	26 (48,1 %)	13 (24,1 %)	2 (3,7 %)
Sicherheit im Umgang mit *Fremd*gefährdung bei *Patient:innen* mit extremistischer Einstellung (*n* = 105)	8 (7,6 %)	39 (37,1 %)	44 (41,9 %)	13 (12,4 %)	1 (1,0 %)
Sicherheit im Umgang mit *Fremd*gefährdung bei *Angehörigen* (*n* = 34)	2 (5,9 %)	6 (17,6 %)	22 (64,7 %)	2 (5,9 %)	2 (5,9 %)

Bei einem Vergleich, ob eher nichtärztliche Psychotherapeut:innen oder Ärzt:innen angaben, mit dem Thema Selbstgefährdung konfrontiert zu sein, zeigte sich, dass vor allem Ärzt:innen solche Situationen erlebten, gleichzeitig aber auch signifikant häufiger Ärzt:innen mit dem Thema Fremdgefährdung konfrontiert waren. Im Umgang mit den Situationen fühlten sich beide Berufsgruppen etwa gleich sicher, durchschnittlich fühlten sich die Fachkräfte „eher nicht“ bis „eher“ sicher (siehe Tab. 3).

**Table 3:** 

	Ärzt:innen *n* = 9–48	Nichtärztliche Psychotherapeut:innen *n* = 20–166		
	*M* (SD)	*M* (SD)	Teststatistik	Eta
*Selbst*gefährdungssituation jemals (*n* = 214)	1,42 (0,50)	1,71 (0,45)	Chi^2^ (1) = 14,070; *p* ≤ 0,001	0,256
*Fremd*gefährdungssituation jemals (*n* = 214)	1,40 (0,49)	1,63 (0,49)	Chi^2^ (1) = 8,106; *p* = 0,005	0,195
Sicherheit im Umgang mit *Selbst*gefährdung bei *Patient:innen* (*n* = 52)	3,05 (0,61)	2,94 (0,67)	Chi^2^ (2) = 0,750; *p* = 0,687	–
Sicherheit im Umgang mit *Selbst*gefährdung bei *Angehörigen* (*n* = 49)	2,88 (1,15)	2,91 (0,91)	Chi^2^ (3) = 0,817; *p* = 0,845	–
Sicherheit im Umgang mit *Fremd*gefährdung bei *Patient:innen* (*n* = 89)	2,41 (0,98)	2,60 (0,94)	Chi^2^ (3) = 1,638; *p* = 0,651	–
Sicherheit im Umgang mit *Fremd*gefährdung bei *Angehörigen* (*n* = 29)	2,44 (1,01)	2,45 (1,15)	Chi^2^ (3) = 2,141; *p* = 0,544	–

Mit einer Gefährdungssituation 1 = „konfrontiert“, 2 = „nicht konfrontiert“

Sicherheit im Umgang mit den Situationen: 1 = „gar nicht“, 2 = „eher nicht“, 3 = „eher“, 4 = „sehr“

Bei einem Vergleich, ob Fachkräfte in Kliniken oder aus Praxen eher angaben, mit dem Thema Selbst- und Fremdgefährdung konfrontiert zu sein, zeigte sich kein signifikanter Unterschied zwischen den beiden Tätigkeitsorten (siehe Tab. 4).

**Table 4:** 

	Klinik *n* = 13–107	Praxis *n* = 18–96		
	*M* (SD)	*M* (SD)	Test-Statistik	Eta
*Selbst*gefährdungssituation jemals (*n* = 202)	1,64 (0,48)	1,59 (0,49)	Chi^2^ (1) = 0,450; *p* = 0,502	–
*Fremd*gefährdungssituation jemals (*n* = 202)	1,53 (0,50)	1,60 (0,49)	Chi^2^ (1) = 1,179; *p* = 0,278	–
Sicherheit im Umgang mit *Selbst*gefährdung bei *Patient:innen* (*n* = 56)	2,89 (0,70)	3,00 (0,54)	Chi^2^ (2) = 2,777; *p* = 0,250	–
Sicherheit im Umgang mit *Selbst*gefährdung bei *Angehörigen* (*n* = 48)	2,67 (1,13)	3,04 (0,91)	Chi^2^ (3) = 1,979; *p* = 0,577	–
Sicherheit im Umgang mit *Fremd*gefährdung bei *Patient:innen* (*n* = 84)	2,54 (0,97)	2,65 (0,77)	Chi^2^ (3) = 0,867; *p* = 0,833	–
Sicherheit im Umgang mit *Fremd*gefährdung bei *Angehörigen* (*n* = 31)	2,31 (1,32)	2,33 (1,09)	Chi^2^ (1) = 5,987; *p* = 0,112	–

Mit einer Gefährdungssituation 1 = „konfrontiert“, 2 = „nicht konfrontiert“

Sicherheit im Umgang mit den Situationen: 1 = „gar nicht“, 2 = „eher nicht“, 3 = „eher“, 4 = „sehr“

### Hinzuziehung von Sicherheitsbehörden

Insgesamt machten 246 Teilnehmende Angaben zu Gefährdungslagen. 50 (20,3 %) der Teilnehmenden mit Angaben zu Gefährdungssituationen gaben an, in den Fällen einer Selbst- und/oder Fremdgefährdung die Sicherheitsbehörden, wie beispielweise die Polizei, kontaktiert zu haben, unabhängig, ob bei Patient:innen mit extremistischer Einstellung oder bei Angehörigen. 28 (63,6 %) von 44 Teilnehmenden gaben an, dass sie den Kontakt mit den Sicherheitsbehörden bei Gefährdungssituationen von Patient:innen mit vermuteter extremistischer Einstellung als hilfreich erlebten. 12 (54,5 %) von 22 Teilnehmenden empfanden den Kontakt im Zusammenhang mit der Behandlung von Angehörigen als eher oder sehr hilfreich in Fällen einer Gefährdung. 10 von 22 Teilnehmenden (45,5 %) empfanden den Kontakt als gar nicht oder eher nicht hilfreich.

Bei einem Vergleich, ob Fachkräfte in Kliniken oder aus Praxen eher angaben, Sicherheitsbehörden hinzugezogen zu haben, zeigte sich kein signifikanter Unterschied (Chi^2^ [1] = 0,001; *p* = 0,979). Bei einem Vergleich, welche der beiden Gruppen (Fachkräfte aus Kliniken, Fachkräfte aus Praxen) den Kontakt mit den Sicherheitsbehörden eher als hilfreich erlebt haben, liegen sowohl bei den Angaben hinsichtlich der behandelten Patient:innen (Chi^2^ [1] = 0,338; *p* = 0,561) als auch hinsichtlich der Angehörigen (Chi^2^ [1] = 0,000; *p* = 1,000) keine signifikanten Gruppenunterschiede vor.

### Zusammenarbeit und Kooperation

Von den insgesamt 247 Teilnehmenden mit Behandlungserfahrungen im Zusammenhang mit Patient:innen mit extremistischer Einstellung und/oder ihren Angehörigen gaben 85 (34,4 %) an, mit externen Stellen kooperiert oder zusammengearbeitet zu haben. 152 (61,5 %) gaben an, dass sie keinen Kontakt mit anderen Stellen im Zusammenhang mit der Krankenbehandlung hatten. 10 Teilnehmende (4,0 %) machten dazu keine Angaben.

Von den 85 Teilnehmenden, welche mit anderen Stellen kooperiert haben, wurde von 9 (10,6 %) Teilnehmenden die Auswahloption gewählt, dass sie im Rahmen der Behandlung Kontakt mit Fachberatungsstellen für Deradikalisierung hatten, 3 (3,5 %) gaben an, Kontakt mit der sogenannten Hotline der Beratungsstelle „Radikalisierung“ des Bundesamts für Migration und Flüchtlinge (BAMF) gehabt zu haben, 14 (16,5 %) hatten Kontakt mit einem Präventionsprojekt bzw. -angebot und 49 (57,6 %) gaben einen Kontakt mit den Sicherheitsbehörden an. Zu sonstigen Stellen hatten 47 (55,3 %) Teilnehmende Kontakt und ergänzten dabei im Freitextfeld z. B. das Jugendamt, Drogenberatungsstellen oder eine ambulante Traumaberatung. Bei der Frage nach spezifischen Kooperationen waren Mehrfachantworten zugelassen.

## Diskussion

Dies ist unseres Wissens nach die erste Studie, die untersucht, ob Ärzt:innen und psychologische Psychotherapeut:innen im Rahmen einer vor allem psychiatrischen oder psychotherapeutischen Behandlung in Kontakt mit Patient:innen mit extremistischer Einstellung und ihren Angehörigen kommen und wie häufig sie dabei mit sicherheitsrelevanten Fragen und Gefährdungssituationen konfrontiert sind. Die Ergebnisse zeigen, dass rund 60 % der Befragten bereits einmal Patient:innen behandelt haben, bei denen sie eine extremistische Einstellung vermutet haben. Bei über der Hälfte der Ärzt:innen und psychologischen Psychotherapeut:innen kam dies mehr als einmal vor. Knapp die Hälfte behandelte zudem schon einmal Angehörige von extremistischen Personen. Bei der Einschätzung, ob Patient:innen eine extremistische Einstellung teilen, sollten sich die Teilnehmenden an der Online-Studie an einer Definition aus der Extremismusforschung orientieren, die darunter ideologische, politische oder religiös motivierte Überzeugungen versteht, die sich außerhalb bzw. am Rande einer freiheitlich-demokratischen Grundordnung bewegen und mit illegalen/gewalttätigen Verhaltensweisen einhergehen können [24]. Durch diese eher eng gefasste Definition sollte vermieden werden, dass Patientinnen und Patienten als extremistisch eingeschätzt werden, die lediglich eine von der Mehrheit der Gesellschaft getragene Meinung nicht teilen.

Die zunächst einmal sehr hohe Anzahl von Fachkräften, die mit dem Thema Extremismus im Rahmen der vor allem psychiatrischen oder psychotherapeutischen Behandlung konfrontiert waren oder aktuell noch sind, ist vorsichtig zu interpretieren. Eine Rolle spielen kann einerseits, dass vor allem diejenigen an der Studie teilgenommen haben, die schon einmal mit dem Thema konfrontiert waren. Motiviert haben könnte die Befragten, dass im Rahmen der Studie vertiefende Fragen zu den Behandlungssituationen beantwortet werden konnten und bei der Werbung der Studie in Aussicht gestellt wurde, dass die Ergebnisse in Schulungsmaterialien einfließen sollen. Andererseits haben gerade die Entwicklungen in den vergangenen Jahren im Zusammenhang mit der COVID-19-Pandemie auch gezeigt, dass vermehrt Arztpraxen mit dem Thema Extremismus konfrontiert wurden und es durchaus auch zu Bedrohungssituationen unmittelbar für Ärztinnen und Ärzte durch extremistische Einstellungen im Rahmen der Behandlung gekommen ist [19]. Auch in der vorliegenden Studie zeigt sich eine vergleichsweise hohe Anzahl von Patientinnen und Patienten, die der „Querdenken“-Bewegung zugeordnet wurden (rund 33 % „Querdenken“-Bewegung vs. beispielweise rund 41 % Rechtsextremismus). Die „Querdenken“-Bewegung stellt hierbei einen neuen Phänomenbereich dar, der im Zusammenhang mit der Pandemie entstanden ist und in Teilen der Bewegung von deutschen Verfassungsschutzbehörden beobachtet wird [20]. An dieser Stelle soll nochmals festgehalten werden, dass es sich bei der Zuordnung zu den Phänomenbereichen um persönliche Einschätzungen der Fachkräfte handelt und keine Einordnung durch die Sicherheitsbehörden.

Gut ein Viertel der Befragten, die Angaben zu Patient:innen machten, berichtete dabei von Situationen der Selbstgefährdung und knapp die Hälfte von Gefährdungssituationen für Dritte, sogenannte Fremdgefährdungssituationen. Diese umfassen beispielsweise von Patient:innen geäußerte Tatpläne oder Hinweise auf eine Gefährdung beispielsweise auch für Kinder oder Lebenspartner:innen. Auch im Zusammenhang mit der Behandlung von Angehörigen wurde sowohl von Selbst- als auch von Fremdgefährdungssituationen berichtet, wobei letztere bei diesen Patient:innen seltener aufgetreten sind als bei unmittelbarer Behandlung von Personen mit extremistischer Einstellung.

In Ergänzung zu den Informationen zu den Gefährdungslagen sollten die Fachkräfte einschätzen, wie sicher sie sich im Umgang damit gefühlt haben. Während mit den Selbstgefährdungssituationen nach eigener Einschätzung eher sicher umgegangen werden konnte, gab weit über die Hälfte an, sich nicht sicher im Umgang mit einer Gefährdung für Dritte gefühlt zu haben. Das mag daran liegen, dass in der Aus- und Weiterbildung das Thema Fremdgefährdung unserer Erfahrung nach eine eher geringere Rolle spielt.

Eine der zentralen Fragen der Studie war es, abzufragen, ob die berichteten Gefährdungslagen zu einer Kontaktaufnahme mit den Sicherheitsbehörden (z. B. Polizei, Landeskriminalamt) geführt haben und ob diese dann als hilfreich wahrgenommen wurde. Etwa 20 % gaben an, Kontakt gehabt zu haben. Über die Hälfte dieser Fachkräfte empfand die Zusammenarbeit dabei als hilfreich, jedoch gab auch knapp die Hälfe an, dass der Kontakt eher oder gar nicht hilfreich war. Die Gründe dafür wurden in dieser Studie nicht erfasst. Wir gehen jedoch davon aus, dass möglicherweise Erwartungen im Zusammenhang mit der gezielten Information der Sicherheitsbehörden, in möglicherweise auch akuten Gefährdungslagen, nicht erfüllt wurden oder es zu Schwierigkeiten bei der Kooperation kam. Dazu muss man wissen, dass die Sicherheitsbehörden ermittelnde Behörden mit dem Ziel der Strafverfolgung sind. Sie gehen zur Abklärung von Gefährdungssituationen daher in der Regel offensiv und nicht zwingend in Absprache mit Behandlerinnen und Behandlern vor. Sollte das Anliegen sein, sich mit der Polizei zu Patientinnen und Patienten hinsichtlich eines Gefährdungspotenzials zunächst beraten zu wollen, dann muss dies im Vorfeld der Kontaktaufnahme unbedingt transparent gemacht werden. Bei Fragen in Fällen, bei denen keine akuten Gefährdungslagen vorliegen, eignen sich für eine Beratung häufig besser sogenannte Fachberatungsstellen für Deradikalisierung, die deutschlandweit speziell Personen mit extremistischer Einstellung und ihre Angehörigen betreuen und in der Regel auch Beratung für Fachkräfte anbieten [29]. Ihre Aufgabe ist es, Menschen bei einem Ausstieg aus dem extremistischen Milieu und/oder auch bei ganz alltäglichen lebenspraktischen Aufgaben zu unterstützen, wie beispielweise bei der Wohnraum- oder Arbeitsplatzsuche. Die zugrunde liegende Hypothese dabei ist, dass Menschen eine extremistische Einstellung aufgrund unterschiedlicher Motivlagen annehmen, die teilweise wenig Verbindung mit der Ideologie haben. Ziel ist es also, Klientinnen und Klienten Lebensperspektiven zu vermitteln und erst im zweiten Schritt im Rahmen eines entstehenden Vertrauensverhältnisses Einstellungen und ideologische Vorstellungen zu diskutieren. In der Untersuchung zeigt sich, dass nur ein kleiner Teil der befragten Fachkräfte bereits mit diesen oder auch anderen Stellen der Extremismusprävention kooperiert. Wir empfehlen Behandlerinnen und Behandler jedoch dringend, diese Stellen zu kontaktieren, um Fragen im Umgang mit den Patient:innen zu klären und um die Patient:innen und ihre Angehörigen an die fachspezifischen Beratungsstellen anzubinden.

### Infobox Deutschlandweite Anlaufstelle zum Thema Extremismus

Beratungsstelle „Radikalisierung“ des Bundesamts für Migration und Flüchtlinge

Hotline +49 911 943 43 43

https://www.bamf.de/DE/Behoerde/Beratungsstelle/beratungsstelle-node.html

Handlungsempfehlung zum Umgang mit extremistischen Einstellungen von Patientinnen und Patienten unter: https://www.uniklinik-ulm.de/fileadmin/default/Kliniken/Kinder-Jugendpsychiatrie/Dokumente/Handlungsempfehlung_Radikalisierungsprozesse.pdf

In einigen Fällen wurden bei den sonstigen zu nennenden Stellen, mit denen bereits kooperiert wird, andere, eher allgemein gehaltene Anlaufstellen genannt, wie das Jugendamt, Drogenberatungsstellen oder eine ambulante Traumaberatung. Dies kann ein Hinweis darauf sein, dass die Probleme von Patient:innen mit extremistischer Einstellung sich vermutlich in vielen Fälle nicht von anderen Situationen in der Krankenbehandlung unterscheiden, wie beispielweise Fällen von Kindeswohlgefährdung [15]. Rund 60 % gaben allerdings auch an, dass sie mit keiner dieser Stellen kooperieren. Möglicherweise waren diesen Fachkräften die genannten Stellen in den Antwortoptionen gar nicht bekannt oder es zeigt sich tatsächlich wenig Bedarf für eine Kooperation. Dabei kann auch eine Rolle spielen, dass Kooperationen zeitintensiv sein können und die Motivation zur Kooperation daher gerade bei niedergelassenen Kolleginnen und Kollegen eher gering ist. Auch im Falle einer Gefährdungssituation gaben gut zwei Drittel an, diese ohne Unterstützung bewältigt zu haben. Dabei gehören Vernetzung und Kooperation zu den bewährten Erfolgsfaktoren in der Extremismusprävention [32]. Vermutlich benötigt es also auch Hilfen und Unterstützung zur Kontaktanbahnung und Vernetzung sowie die Verdeutlichung von Vorteilen einer Kooperation. Denn längst wird beispielweise vonseiten der Fachberatungsstellen für Deradikalisierung gefordert, dass eine Zusammenarbeit mit Fachkräften aus den Heilberufen aus der Sicht der Fachberatung dringend notwendig ist [32], da sich dort vermehrt Klientinnen und Klienten mit psychischen Auffälligkeiten befinden [32]. Auch wenn die Befundlage zu psychischen Erkrankungen bei diesen Menschen noch wenig aussagekräftig ist (siehe hierzu die Literatur in der Einleitung; [25, 31]), sollte zumindest immer eine psychiatrische Abklärung möglicher Erkrankungen erfolgen – nicht zuletzt, um das Risiko für Gewalttaten zu senken.

Noch einmal möchten wir erwähnen, dass der Kontakt mit den Sicherheitsbehörden vor allem dann gefragt ist, wenn es sich um wesentliche Aspekte der Gefahrenabwehr und des Opferschutzes handelt. Um festzuhalten, inwieweit die Zusammenarbeit von Polizei, Landeskriminalämtern und Verfassungsschutz mit Heilberufen optimiert werden kann, da sich eben nicht alle Kontakte mit den Sicherheitsbehörden als hilfreich in dieser Untersuchung gezeigt haben, ist es ein Anliegen der Autorinnen und Autoren der Studie, diese Fragestellung weiter zu untersuchen.

In der Auswertung der Daten zeigt sich darüber hinaus, dass Ärzt:innen nach eigenen Angaben signifikant häufiger mit Fremd- und Selbstgefährdungssituationen konfrontiert waren als nichtärztliche Psychotherapeut:innen, während es in Bezug auf die Behandlung in einer Praxis im Gegensatz zu einer klinischen Arbeit keine Unterschiede gab und sich auch keine Unterschiede der beiden Gruppen zeigten bezüglich der Kontaktaufnahme mit den Sicherheitsbehörden in Gefährdungslagen. Ein Grund für die stärkere Wahrnehmung von Gefährdungssituationen bei Ärztinnen und Ärzten könnte sein, dass sie solche Situationen eher auch als solche wahrgenommen haben oder Patientinnen und Patienten in psychiatrischer Behandlung mehr psychopathologische Auffälligkeiten zeigen oder insgesamt schwerer psychisch erkrankt sind und es somit auch häufiger zu Gefährdungssituationen kam [12, 23, 33].

Abschließend ist zu bedenken, dass es sich bei der Untersuchung um eine erste Studie in diesem Themenfeld handelt. Sie ist keine repräsentative Befragung, sodass damit lediglich ein Ausschnitt aus der psychiatrischen und psychotherapeutischen Praxis berichtet wird. Dabei ist, wie bereits erwähnt, davon auszugehen, dass vor allem diejenigen an der Befragung teilgenommen haben könnten, die bereits mit dem Thema innerhalb der Krankenbehandlung konfrontiert waren, Erfahrungen sammeln konnten oder sich speziell für dieses Thema interessieren.

## Fazit für die Praxis

- Mit der vorliegenden Studie wurde aufgezeigt, dass extremistische Einstellungen ein Thema in der Krankenbehandlung sind und es in diesem Zusammenhang auch zu Gefährdungslagen kommt, auf die Ärzt:innen und Psychotherapeut:innen gut vorbereitet sein sollten. Eine Hilfe könnte eine bessere Vernetzung mit Angeboten der Extremismusprävention sein, aber auch eine gute Zusammenarbeit mit Sicherheitsbehörden. Dazu benötigt es
  - Unterstützung im Bereich Vernetzung und Kooperation,
  - Wissen über Ansprechpersonen bei (akuten) Gefährdungslagen und
  - weitere Analysen zur Optimierung der Kooperation mit Sicherheitsbehörden.
